# Elevated N-terminal pro-brain natriuretic peptide is associated with increased mortality in patients with COVID-19: systematic review and meta-analysis

**DOI:** 10.1136/postgradmedj-2020-137884

**Published:** 2020-05-20

**Authors:** Raymond Pranata, Ian Huang, Antonia Anna Lukito, Sunu Budhi Raharjo

**Affiliations:** Faculty of Medicine, Universitas Pelita Harapan, Tangerang, Indonesia; Faculty of Medicine, Universitas Pelita Harapan, Tangerang, Indonesia; Department of Internal Medicine, Hasan Sadikin General Hospital-Faculty of Medicine, Universitas Padjadjaran, Bandung, Indonesia; Faculty of Medicine, Universitas Pelita Harapan, Tangerang, Indonesia; Cardiology and Vascular Medicine, Siloam Hospitals Lippo Village, Tangerang, Indonesia; Cardiology and Vascular Medicine, Faculty of Medicine Universitas Indonesia, National Cardiovascular Center Harapan Kita, Jakarta, Indonesia

**Keywords:** intensive & critical care, infectious diseases, respiratory infections

## Abstract

**Objectives:**

This systematic review and meta-analysis aimed to assess the association between N-terminal pro-brain natriuretic peptide (NT-proBNP) and mortality in patients with COVID-19.

**Methods:**

Systematic literature search from several electronic databases were performed. The outcome was mortality (non-survivor) in patients with COVID-19 pneumonia. NT-proBNP data were in continuous variable (pg/mL), dichotomous data (elevated/non-elevated) and effect estimate adjusted to cardiac injury/elevated biomarkers of cardiac injury.

**Results:**

A total of 967 patients from six studies were included in this analysis. NT-proBNP was higher in non-survivor group (standardised mean difference 0.75 (0.44, 1.07), p<0.001; I^2^: 61%). Elevated NT-proBNP was associated with increased mortality (RR 3.63 (92.21, 5.95), p<0.001; I^2^: 60%). Sensitivity analysis by removing a study reduces heterogeneity (risk ratio 3.47 (2.36, 5.11), p<0.001; I^2^: 49%). Pooled adjusted HR (adjusted to cardiac injury/elevated biomarkers of cardiac injury) showed that elevated NT-proBNP was independently associated with mortality (HR 1.37 (1.19, 1.57), p<0.001; I^2^: 0%, p=0.77). Pooled analysis of multiple cut-off point resulted in a sensitivity of 76% (46%–92%) and specificity of 88% (71%–96%). Summary receiver operating characteristic curve analysis demonstrates an area under curve of 0.90 (0.87–0.93). Elevated NT-proBNP has a likelihood ratio (LR) +6.4 and LR -0.3.

**Conclusion:**

Elevated NT-proBNP level was associated with increased mortality in COVID-19 pneumonia.

## Introduction

The World Health Organization (WHO) affirmed Coronavirus Disease 2019 (COVID-19) as a public health emergency of international concern and declared it as a pandemic on 11 March 2020.^1^ Globally, there were more than 1 800 000 people infected by COVID-19 which resulted in 110 000 deaths.^2^ While patients with COVID-19 commonly have mild symptoms or even be asymptomatic, a notable proportion of patients may develop severe pneumonia, acute respiratory distress syndrome (ARDS), multiorgan failure and, death.^3^ Markers to risk-stratify patients with COVID-19 are crucial during a pandemic in which resource allocation needs to be judiciously organized. ^4^

Cardiac injury is present in up to 20% of hospitalised patients with COVID-19.^5^ A recently published study showed that N-terminal pro-brain natriuretic peptide (NT-proBNP) increases the risk of mortality in patients with COVID-19.^6^ NT-proBNP is a natriuretic peptide released as a response to increased ventricular wall tension, it is a marker of reduced left ventricular systolic function and poor prognosis in patients with heart failure.^7  8^ In this systematic review and meta-analysis, we aimed to assess the association between NT-proBNP and mortality in patients with COVID-19.

## Methods

### Search strategy and study selection

We performed a comprehensive systematic literature search from PubMed, SCOPUS, EuropePMC, Cochrane Central Databases with the search terms: (1) ‘COVID-19’ OR ‘SARS-CoV-2’ AND ‘Cardiac’, (2) ‘COVID-19’ OR ‘SARS-CoV-2’ AND ‘Characteristic’, we limit the search results to the year 2020. After initial search, the duplicate results were then removed. Two independent authors (IH and RP) performed screening of the abstracts and title for potential articles. Full texts of potential articles were then assessed by applying inclusion and exclusion criteria. We finalised the search on 8 April 2020.

### Inclusion and exclusion criteria

Original articles containing data on NT-proBNP and its association with mortality in patients with COVID-19 were included in this systematic review and meta-analysis. Review articles, case reports, letter to editor and correspondence that did not report primary data were excluded from the analysis. Articles in non-English language were also excluded.

### Data extraction

Two independent authors (IH and RP) performed data extraction. To facilitate data extraction, we used a standardised extraction forms containing authors in the rows and year, study design, gender, age, NT-proBNP level (and its cut-off point), troponin level, age, gender, hypertension, coronary artery/cardiovascular diseases, respiratory comorbidities and mortality in the column.

The outcome of interest was mortality (non-survivor) in patients with COVID-19 pneumonia. NT-proBNP data were in continuous variable (pg/mL), dichotomous data (elevated/non-elevated) and effect estimate adjusted to cardiac injury/elevated biomarkers of cardiac injury.

### Statistical analysis

To perform meta-analysis, we used Review Manager V.5.3 (Cochrane Collaboration) and Stata V.16. We used the inverse-variance method to assess continuous variables and the pooled effect estimate was reported as standardised mean differences (SMD) with its SD. Mantel-Haenszel formula was used for dichotomous variables to calculate the risk ratios (RRs) and its 95% CIs. The pooling of adjusted effect estimate was done using inverse-variance formula to calculate HR which was reported along its 95% CIs. All p values in this meta-analysis were two-tailed, and the statistical significance was set at ≤0.05 (except for heterogeneity, which is <0.10). Leave-one-out sensitivity analysis was performed to assess the cause of heterogeneity. To assess the risk of publication bias qualitatively, inverted funnel-plot analysis was performed. Regression-based Egger’s and Harbord’s test were then performed to assess the small-study effect for continuous variable and dichotomous variable, respectively.

## Results

### Baseline characteristics and study selection

**Figure 1:**
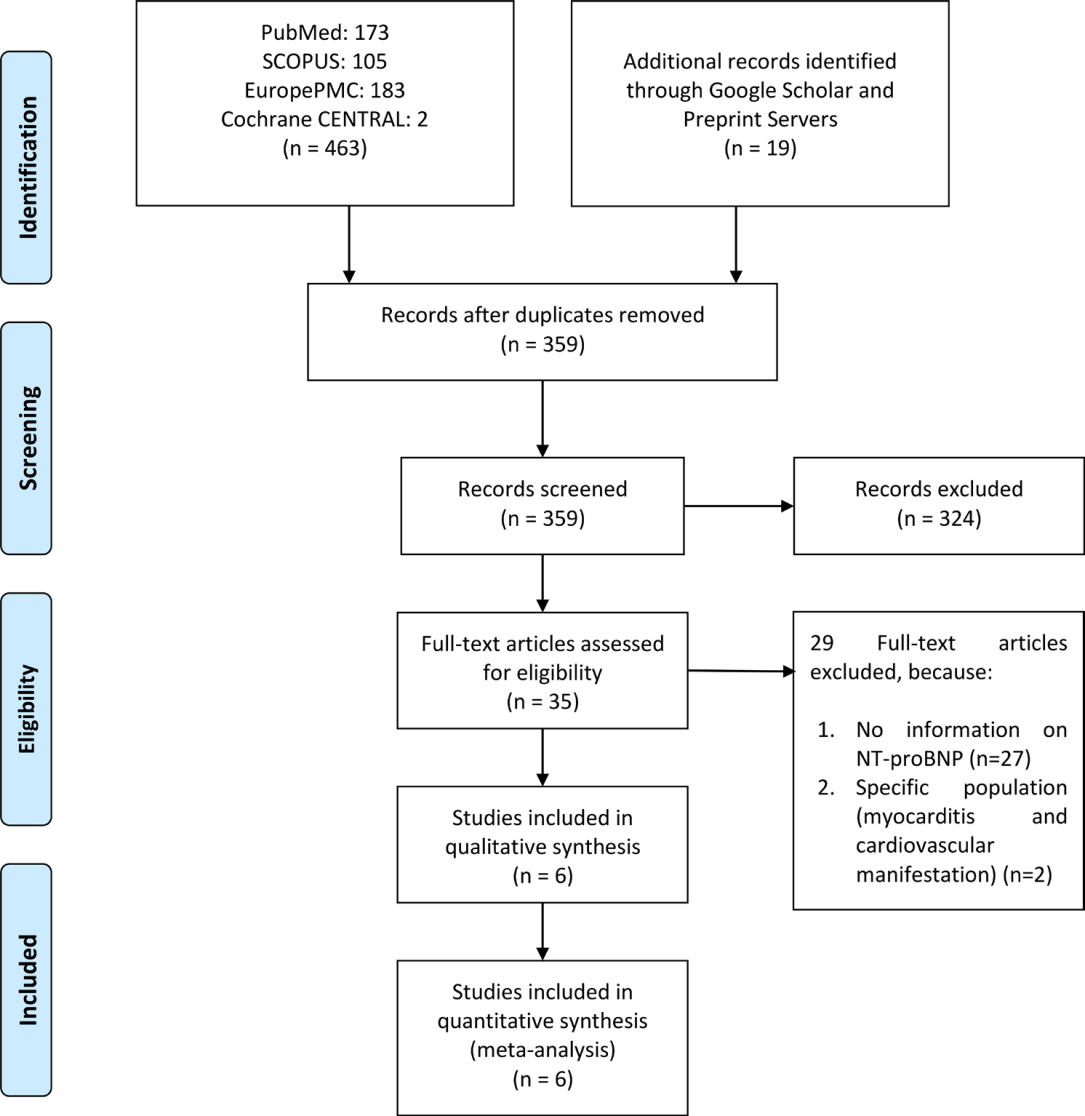
Preferred Reporting Items for Systematic Reviews and Meta-Analyses study flow diagram. NT-proBNP, N-terminal pro-brain natriuretic peptide.

Initial search yields 482 records, and after screening+duplicate removal, 359 records remained. Title and abstracts were then screened to identify potential studies, in which 324 records were removed. Thirty-five full texts were then assessed for eligibility, and a total of 29 were excluded because of (1) no information on NT-proBNP (n=27) and (2) specific study population (myocarditis and patients with cardiovascular manifestation) (n=2). There were six studies eligible for qualitative and quantitative synthesis^5  6  9–12^ (figure 1). There were a total of 967 patients from six studies. All of the included studies were retrospective observational in design. Table 1 shows the baseline characteristics of the included studies.

**Table 1:** Characteristics of the included studies

Authors	Study design	Samples	BNP	Cut-off	Mean/median hs-cTnI (non-survivor vs survivor) (pg/mL)	Male (%)	Mean/median Age (years)	HTN (%)	CAD/CVD (%)	Diabetes (%)	Respiratory comorbidities (%)
Cao J 2020	Observational, Retrospective	102 (17/85)	NT-pro BNP	≥900 pg/mL	21.5 (9.4–44.1) vs 7.6 (3.2–11.0) (All)	76.5 vs 47.1	72 vs 53	64.7 vs 20	17.6 vs 2.4 (CAD)	35.3 vs 5.9	23.5 vs 7.1 (COPD)
Chen 2020^3^	Observational, Retrospective	123 (31/92)	NT-pro BNP	N/A	0.21±0.45 vs 0.01±0.01 *troponin T	71 vs 42	72 vs 53	48.4 vs 38.3	25.8 vs 7.6 (CAD)	19.4 vs 8.7	9.7 vs 3.3 (COPD)
Chen T 2020^6^	Observational, Retrospective	274 (113/161)	NT-pro BNP	≥285 pg/mL	40.8 (14.7–157.8) vs 3.3 (1.9–7.0)	73 vs 55)	68.0 vs 51.0	48 vs 24	14 vs 4 (CVD)	21 vs 14	10 vs 4(CLD)
Li K 2020^9^	Observational, Retrospective	32 (11/21)	NT-pro BNP	≥241 pg/mL	24.1 (13.0–202.1) vs 4.3 (2.0–10.6)	73 vs 55)	57 (69 vs 55)	30 (47 vs 28)	4 (13 vs 2) (CAD)	15 (13 vs 15)	2 (7 vs 1) (COPD)
Gao L 2020*^12^	Observational, Retrospective	54	NT-pro BNP	>88.64 pg/mL	N/A	N/A	N/A	N/A	N/A	N/A	N/A
Shi S 2020*^5^	Observational, Retrospective	416	NT-pro BNP	N/A	N/A	N/A	N/A	N/A	N/A	N/A	N/A

*Group was not mortality versus no mortality (high NT-proBNP vs low-moderate NT-proBNP; cardiac injury vs no cardiac injury).

CAD, coronary artery disease; CLD, Chronic Lung Disease; COVID-19, coronavirus disease 2019; CVD, cardiovascular Disease; HTN, hypertension; N/A, not available; NT-proBNP, N-terminal pro-brain natriuretic peptide.

### Association between NT-proBNP and mortality in COVID-19

**Figure 2:**
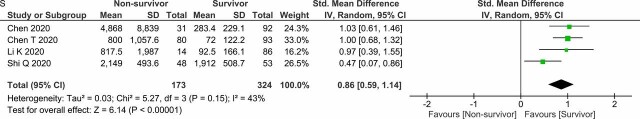
NT-proBNP concentration and mortality. Non-survivors have a higher concentration of NT-proBNP compared with survivors. NT-proBNP, N-terminal pro-brain natriuretic peptide.

**Figure 3:**
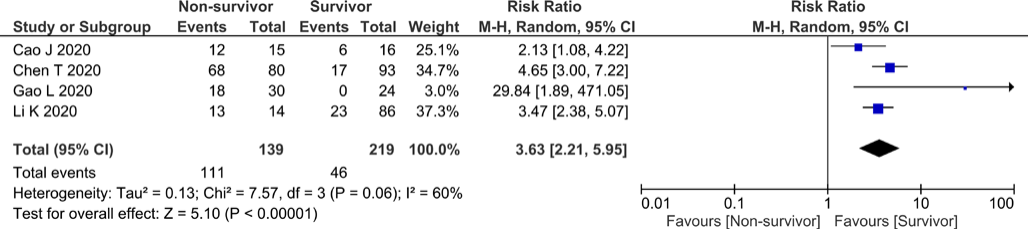
Elevated NT-proBNP and mortality. Elevated NT-proBNP was associated with increased mortality. NT-proBNP, N-terminal pro-brain natriuretic peptide.

**Figure 4:**
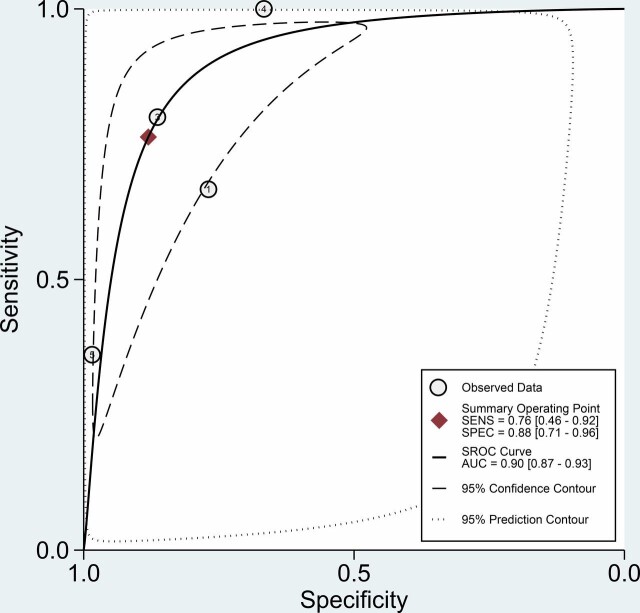
SROC curve for elevated NT-proBNP and mortality. SROC curve for pooled analysis of elevated NT-proBNP at multiple cut-off points. NT-proBNP, N-terminal pro-brain natriuretic peptide. SROC: Summary receiver operating characteristic

Meta-analysis showed that NT-proBNP was higher in non-survivor group (SMD 0.75 (0.44, 1.07), p<0.001; I^2^: 61%, p=0.04) (figure 2). Elevated NT-proBNP was associated with increased mortality (RR 3.63 (2.21, 5.95), p<0.001; I^2^: 60%, p=0.06) (figure 3). Sensitivity analysis by removing Gao *et al*’s study showed that heterogeneity could be reduced (RR 3.47 (2.36, 5.11), p<0.001; I^2^: 49%, p=0.14). Pooled analysis of multiple cut-off point resulted in a sensitivity of 76% (46%–92%) and specificity of 88% (71%–96%). Summary receiver operating characteristic (SROC) curve analysis (with prediction and confidence contours) demonstrate an area under curve (AUC) of 0.90 (0.87–0.93) (figure 4). Elevated NT-proBNP has a likelihood ratio (LR) +6.4 and LR -0.3. Pooled adjusted HR (adjusted to cardiac injury/elevated biomarkers of cardiac injury) showed that elevated NT-proBNP was independently associated with mortality (HR 1.37 (1.19, 1.57), p<0.001; I^2^: 0%, p=0.77).

### Risk of publication bias

**Figure 5:**
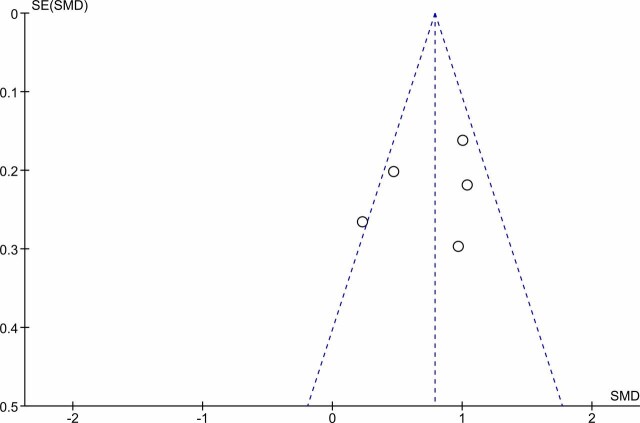
Funnel-plot analysis. Funnel-plot analysis showing asymmetrical funnel plot indicating possible risk of publication bias. SMD, standardised mean difference.

Inverted funnel-plot analysis demonstrated a qualitatively asymmetrical shape, which indicates the possibility of publication bias (figure 5). Regression-based Harbord’s test was statistically significant for small-study effects (p=0.034). Egger’s test indicates a statistically significant small-study effects for the continuous variable (p=0.002).

## Discussion

This meta-analysis showed elevated NT-proBNP level was associated with increased mortality in COVID-19 pneumonia with satisfying AUC and specificity.

NT-proBNP has been shown to predict short-term and long-term mortality in patients with pneumonia.^13–15^ Critically ill patients with pneumonia have elevated NT-proBNP concentration in the intensive care unit setting.^14^ Hence, NT-proBNP can be used for the risk-stratification purpose in patients without chronic heart failure. Indeed, NT-proBNP has also been shown to be a marker of poor prognosis in patients with sepsis and ARDS.^16  17^ Hypoxia-induced pulmonary hypertension in patients with pneumonia may increase ventricular wall stress and leads to the release of NT-proBNP.^18^ The use of vasopressor in critically ill patients may also contribute further to the wall stress.^14^ Presence of renal failure in critically ill patients may also impair NT-proBNP clearance.^19  20^ Pneumonia is postulated to cause relative ischaemia, sympathetic upregulation, systemic inflammation and direct pathogen-mediated damage to the cardiovascular system.^18^ Furthermore, pneumonia has been shown to increase short-term and long-term risk of cardiovascular disease, bridging the aforementioned hypothesis.^21^ A similar mechanism may underlie NT-proBNP elevation in patients with severe COVID-19 pneumonia; this meta-analysis only demonstrates the short-term outcome, follow-up is needed for the longer term outcome. NT-proBNP is postulated to increase the risk of heart failure in patients with COVID-19.^22^

A meta-analysis showed that elevated troponin increased the risk for mortality and become a possible confounder in the analysis.^23^ Nevertheless, the current meta-analysis also showed the possibility that NT-proBNP was independently associated with mortality after adjustment to troponin and creatine kinase myocardial band.

### Implications for clinical practice

NT-proBNP may be used for risk stratification of patients with COVID-19 in order to determine treatment strategies based on risk in a tight resource situation due to pandemic. We encourage studies that aim to develop the prognostication model to include NT-proBNP as one of the biomarkers in their study.

### Limitation

The limitation of this systematic review and meta-analysis is the presence of publication bias, as indicated funnel plot, Egger’s and Harbord’s test. The sample size was also small; due to the novelty of the virus, the report on NT-proBNP was scarce. Furthermore, the cut-off points differ across the studies. The articles included in this meta-analysis were mostly preprints; nevertheless, exhaustive efforts have been made to ensure that only sound studies were included in the analysis.

## Conclusions

This meta-analysis showed elevated NT-proBNP level was associated with increased mortality in COVID-19 pneumonia.

**Main messages**

- Level of N-terminal pro-brain natriuretic peptide (NT-proBNP) was higher in non-survivor group.
- NT-proBNP was associated with mortality in both pooled unadjusted and adjusted models.
- It has 76% sensitivity and 88% specificity, and area under curve of 0.90.

**Current research questions**

- An effective prognostication model remains to be explored in patients with COVID-19.
- Prospective studies with larger sample size and similar/uniform cut-off points are needed to confirm this finding.
- Studies outside China are needed to make conclusion more generalisable.

**What is already known on the subject**

- Cardiac injury is present in up to 20% of hospitalised patients with COVID-19.
- NT-proBNP has been previously shown as a goodreliable prognostic marker in patients with pneumonia
